# Stromal Versican Regulates Tumor Growth by Promoting Angiogenesis

**DOI:** 10.1038/s41598-017-17613-6

**Published:** 2017-12-08

**Authors:** Keiichi Asano, Courtney M. Nelson, Sumeda Nandadasa, Noriko Aramaki-Hattori, Daniel J. Lindner, Tyler Alban, Junko Inagaki, Takashi Ohtsuki, Toshitaka Oohashi, Suneel S. Apte, Satoshi Hirohata

**Affiliations:** 1 Department of Molecular Biology and Biochemistry, Okayama University Graduate School of Medicine, Dentistry and Pharmaceutical Sciences, 2-5-1, Shikata-cho, Okayama, Japan; 2 Department of Biomedical Engineering, Cleveland Clinic Lerner Research Institute, Cleveland, OH USA; 3 Translational Hematology & Oncology Research, Cleveland Clinic Taussig Cancer Center, Cleveland, OH USA; 4 Department of Cell Chemistry, Okayama University Graduate School of Medicine, Dentistry and Pharmaceutical Sciences, 2-5-1, Shikata-cho, Okayama, Japan; 5 Department of Medical Technology, Graduate School of Health Sciences, Okayama University, 2-5-1, Shikata-cho, Okayama, Japan

## Abstract

The proteoglycan versican is implicated in growth and metastases of several cancers. Here we investigated a potential contribution of stromal versican to tumor growth and angiogenesis. We initially determined versican expression by several cancer cell lines. Among these, MDA-MB231 and B16F10 had none to minimal expression in contrast to Lewis lung carcinoma (LLC). Notably, tumors arising from these cell lines had higher versican levels than the cell lines themselves suggesting a contribution from the host-derived tumor stroma. In LLC-derived tumors, both the tumor and stroma expressed versican at high levels. Thus, tumor stroma can make a significant contribution to tumor versican content. Versican localized preferentially to the vicinity of tumor vasculature and macrophages in the tumor. However, an ADAMTS protease-generated versican fragment uniquely localized to vascular endothelium. To specifically determine the impact of host/stroma-derived versican we therefore compared growth of tumors from B16F10 cells, which produced littleversican, in *Vcan* ^*hdf/*+^ mice and wild-type littermates. Tumors in *Vcan* ^*hdf/*+^ mice had reduced growth with a lower capillary density and accumulation of capillaries at the tumor periphery. These findings illustrate the variability of tumor cell line expression of versican, and demonstrate that versican is consistently contributed by the stromal tissue, where it contributes to tumor angiogenesis.

## Introduction

Angiogenesis, the formation of neovasculature from pre-existing capillaries, is a crucial event during malignant tumor progression. It provides oxygen and nutrients to the growing tumor and a pathway for tumor cell metastasis to distant organs^1,2^. Tumor growth and angiogenesis occur within a complex host/stromal microenvironment comprising vascular endothelium, immune cells, fibroblasts and other cell types. The tumor also contains an extracellular matrix (ECM), which may be contributed by the stromal or tumor cells. It includes proteoglycans, hyaluronan (HA), collagens, and a variety of glycoproteins. The mechanisms by which this tumor microenvironment governs tumor angiogenesis is poorly understood and is a promising area for new therapeutic strategies^3^.

Versican is a large chondroitin sulfate proteoglycan that forms aggregates with HA which connects it to the cell surface via HA receptors such as CD44^4–6^. Versican is implicated in many biological processes involving the vasculature, such as atherosclerosis and vascular inflammation^7,8^. There are five known versican splice isoforms; V0, V1, V2, V3 (Supplementary Fig. S1A) and V4^9^. Each isoform except V3 has a glycosaminoglycan (GAG) domain with covalently attached chondroitin sulfate (CS) chains between the globular domains 1 (G1) and G3 (Supplementary Fig. S1B). Of these, V2 is not generally seen outside the nervous system. In addition to HA-binding via G1, the versican GAG-chains and G3 domain have multiple intermolecular interactions^10,11^. Versican and its recombinant domains are implicated in regulating cell proliferation, differentiation, apoptosis, migration and adhesion in a variety of cancers^12,13^. Abundant evidence suggests that versican is anti-adhesive, since it is a poor cell attachment and migration substrate^14,15^ and is excluded from focal adhesions^16–18^. Several clinical studies have suggested that high versican expression is a poor prognostic factor in a variety of cancers^19–24^.

Several members of the family of A disintegrin-like and metalloproteinase with thrombospondin type 1 motifs (ADAMTS) are involved in versican proteolysis and tumor progression^25–29^. ADAMTS-1, -4, -5, -9, -15 and -20 cleave versican at a specific site in the GAGβ region and ADAMTS4 has been shown to cleave an additional, poorly characterized site in the GAGα domain (Supplementary Fig. S1C)^9^. Versican turnover by ADAMTS proteases is well-characterized during embryogenesis and accompanies discrete morphogenetic events^30–32^. ADAMTS proteolysis of the V1 isoform generates an N-terminal G1 domain-containing fragment named versikine, which was implicated in regulation of apoptosis during limb development and myeloma growth^33^. Previous studies suggested that versican cleavage by ADAMTS proteases also occurs during vascular remodeling^34–37^, in human uterine leiomyoma^38^ and in human myeloma^33^. However, the spatial and causal relationship between versican, versican cleavage and tumor angiogenesis remains incompletely understood^39,40^. Here, we investigated the contribution of stromal production of versican and of versican cleavage to tumor angiogenesis.

## Results

### Tumor cell lines show varying levels of versican expression

**Figure 1:**
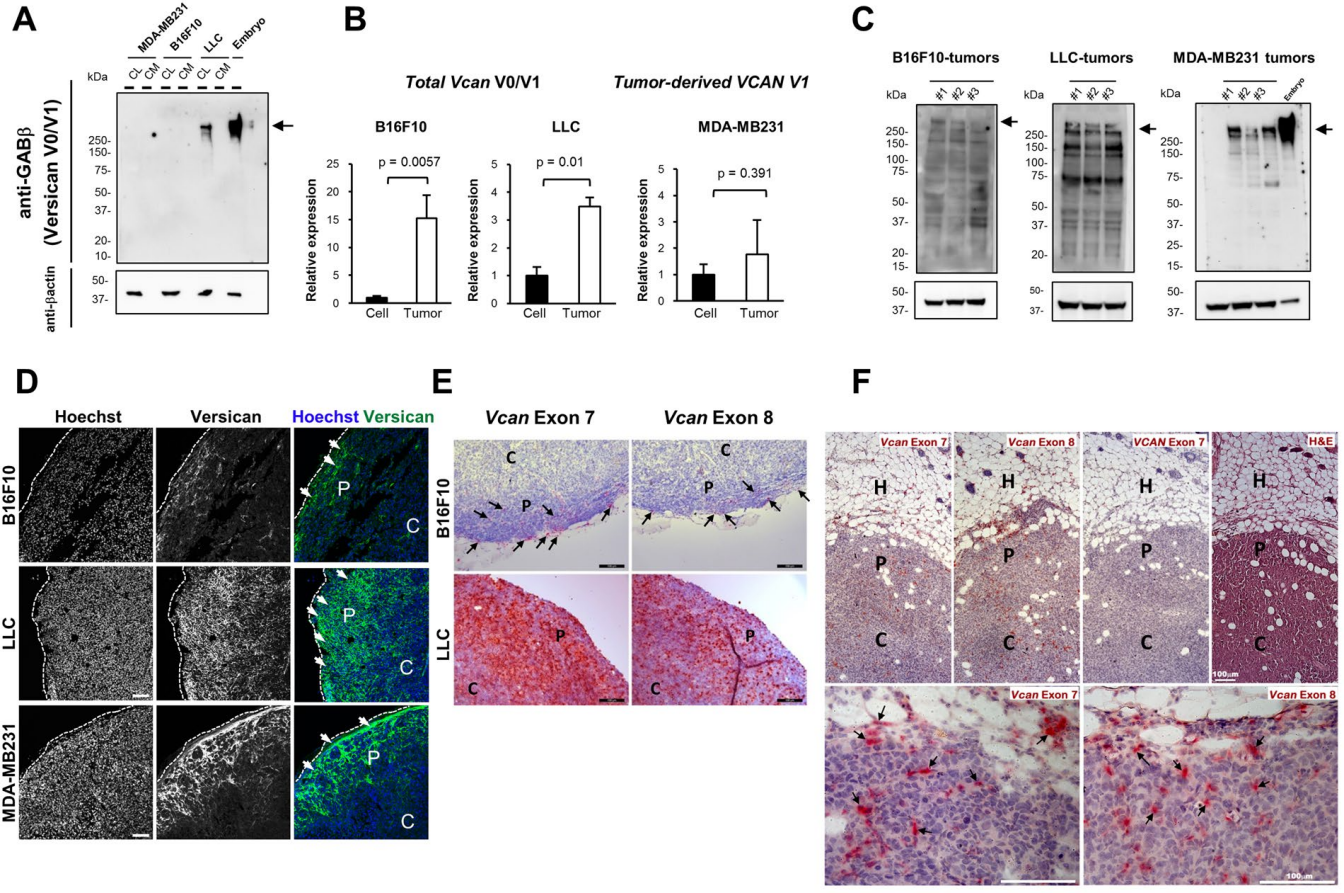
Expression, distribution and origin of versican GAGβ in cancer cell lines and tumor tissues. (**A**) Protein production of versican GAGβ in conditioned medium (CM) obtained from MDA-MB231, B16F10 and Lewis lung carcinoma (LLC) cancer cell lines and cell lysates (CL) was determined by Western blot analysis with anti-GAGβ antibody. Protein extract from an E13.5 mouse embryo (indicated as Embryo) was used as a positive control. Anti-β-actin antibody was used to indicate protein loading for cell lysates. Arrows indicate the predicted size of versican. (**B**) Expression of mouse versican V0 and/or V1 (*Vcan* V0/V1) and human versican V1 (*VCAN* V1) mRNA in cancer cells (cell) and corresponding tumors (tumor) was compared by quantitative reverse transcription PCR (qPCR). Each expression was normalized to *Gapdh* (for mouse versican) and *RPLP2* (for human versican) mRNA levels. Please note that *VCAN* V1 RT-PCR determined the expression of tumor-derived *VCAN* V1 (human) in the MDA-MB231 xenograft tumors since they contain human-derived tumor cells and mouse-derived host cells. n = 4 for B16F10; n = 3 for LLC; n = 3 for MDA-MB231. Statistical significance was evaluated by two-tailed unpaired t-test. (**C**) Versican GAGβ content in B16F10-, LLC- and MDA-MB231-derived tumors was determined by Western blotting using anti-versican GAGβ antibody. Protein extract from E13.5 mouse embryo was used as the positive control. Arrows indicate the predicted size of versican. Note extensive versican proteolysis in all blots. (**D**) Versican GAGβ immunostaining (green) in B16F10- (upper panel), LLC- (middle panel) and MDA-MB231- (lower panel) tumors. Nuclei were counterstained with Hoechst dye (blue). The white dashed lines indicate the edge of each tumor. C: center of the tumor; P: periphery of the tumor. Arrows indicate the positive signal (green) in the tumors. Scale bars = 100 μm (**E**) *RNA in situ* hybridization (red signal) of mouse *Vcan* exon 7 (mouse *Vcan* V0 and/or V2) and exon 8 (mouse *Vcan* V0 and/or V1) in B16F10- (upper panels) and LLC-tumors (lower panels). Please note that both large exons of versican (i.e., exon 7 (*Vcan* V0 and/or V2) and exon 8 (*Vcan* V0 and/or V1)) were expressed in the tumor periphery. C: center; P: periphery. Arrows indicate the positive signals in B16F10-tumor. (**F**) *In situ* hybridization using mouse *Vcan* exon 7 (mouse V0/V2) and exon 8 (mouse V0/V1) to identify mouse-derived versican mRNA and human *VCAN* exon 7 (human V0/V2) in the MDA-MB231 xenograft tumor. The panel at right shows hematoxylin and eosin stain. Arrows indicate the positive signals in MDA-MB231 tumor. Please note that the positive signals indicate the versican expressing cell-derived from host (mouse) cells. Scale bars = 50 μm for *in situ* hybridization and 100 μm for hematoxyline-eosin staining. H, host tissue; P: periphery; C: center.

Versican mRNA and protein levels have not been systematically compared in experimentally used cancer cell lines and tumors arising from them. We first examined the expression of splice variants V0, V1, and V2 (Supplementary Fig. S2) in commonly used cancer cell lines. By reverse transcription PCR (RT-PCR) and quantitative RT-PCR (qPCR), minimal versican V0, V1 and V2 mRNA expression was seen in MDA-MB231- cells relative to human skin fibroblasts (Supplementary Fig. S2A,B). Similarly, B16F10- cells did not express versican, whereas LLC cells showed high levels of *Vcan* V0 and V1 mRNA comparable to the aorta (Supplementary Fig. S2C,D). Western blotting confirmed a lack of versican GAGβ, representing isoforms V0/V1 in MDA-MB231- and B16F10-cells compared to LLC cells and mouse embryo lysate as a positive control (Fig. 1A). Similarly, RT-PCR and qPCR for other cancer cell lines, MS-1, 4T-1, and SVR showed very low expression, while TRAMP-C1, TRAMP-C2 and FCB cell lines showed high levels of versican, comparable to mouse skin fibroblasts and mouse embryonic fibroblasts which are known for robust versican expression (data not shown). Thus, various cancer cell lines differ widely in their versican expression levels.

### The tumor microenvironment contributes versican production

We next examined the contribution of stroma- derived versican in the tumor microenvironment. Based on the above finding of negligible versican production by B16F10- and MDA-MB231 cells, we selected them for subsequent analysis. We compared versican mRNA expression and content in the B16F10 and MDA-MB231 cells versus the respective tumors obtained by their injection into mice. *Vcan* V0/V1 mRNA was significantly higher in the B16F10 tumors than in cultured B16F10 cells (Fig. 1B). *VCAN* V1 mRNA in MDA-MB231 cells and tumors were comparable (Fig. 1B). In B16F10 tumors, both the host- and tumor derived versican would be of mouse origin and q-PCR detected the cumulative levels of mouse *Vcan* mRNA in tumor and host. In MDA-MB231 cells, q-PCR specifically detected human mRNA. Furthermore, even in tumors arising from LLC cells, which express versican robustly, we observed increased *Vcan* mRNA levels, suggestive of a contribution of tumor microenvironment (i.e., stroma)(Fig. 1B).

Because we did not specifically assess host contribution in MDA-MB231 tumors by q-PCR, we selected to perform *in situ* hybridization using species-specific probes (see below). In contrast to Western blots of the cell lines (Fig. 1A), the tumor Western blots showed abundant versican, although mostly in a variety of cleaved forms (Fig. 1C). It should be noted that versican GAGβ antibody detected both mouse and human versican. These results suggested that the tumor microenvironment itself may contribute versican production or could potentially induce *de-novo* versican expression in tumor cells that do not express versican in culture. They also indicated extensive proteolysis of versican in the tumor microenvironment. Immunostaining for versican GAGβ showed specific staining at the periphery of B16F10-, LLC- and MDA-MB231-tumors, with the LLC tumors showing also strong staining in the tumor center (Fig. 1D). The specificity of the versican GAGβ antibody was validated by staining embryonic mouse limbs^34^ (Supplementary Fig. S3A). *In situ* hybridization with mouse probes demonstrated that *Vcan* exon 7 and exon 8, encoding versican GAGα and GAGβ domains, were both expressed at the tumor periphery in B16F10-derived tumors (Fig. 1E). In contrast, the tumors derived from LLC-cells showed strong expression throughout the tumor (Fig. 1E). These findings are consistent with immunostaining (Fig. 1D) and suggest that in B16F10-derived tumors, versican arises from stromal cells invading from the periphery of the tumor. In LLC-derived tumors, versican was produced from both tumor and stromal cells. We next sought to determine the origin of versican in the MDA-MB231 tumors, which contain both human tumor cells and stromal cells of mouse origin. We performed *in situ* hybridization using probes designed against the human *VCAN* exon 7 or mouse *Vcan* exon 7 and 8. *Vcan* (mouse) probes specific for exon 7 and exon 8 detected positive signals in E16.5 mouse embryo sections (Supplementary Fig. S4A–C) but not human umbilical cord (Supplementary Fig. S4F). *VCAN* (human) exon 7 and exon 8-specific probes detected positive signals in human umbilical cord but not in mouse embryos (Supplementary Fig. S4D–F), demonstrating stringent species-specificity. With these species-specific probes, we observed positive signal for *Vcan* exon 7 and exon 8 in the MDA-MB231 xenograft tumors. The signal was strongest in the periphery of the tumor, especially over the bordering host/stromal tissue (Fig. 1F). The majority of the tumor section, including large areas of tumor cells were negative. Together with the specificity of the mouse probe, this suggests that versican present in MDA-MB231 tumors originates from host-derived stromal cells. The precise stromal cell phenotype expressing the mRNA cannot be readily ascertained owing to the challenges of combining immunostaining with *in situ* hybridization on the same section. The *VCAN* exon 7 probe (human), gave no signal in the MDA-MB231 xenograft tumors (Fig. 1F). Thus, these observations clearly indicate that tumor microenvironment consistently contributes versican production to the tumors arising from these different cell lines.

### Host-derived versican is located in vasculature and in the vicinity of tumor macrophages

**Figure 2:**
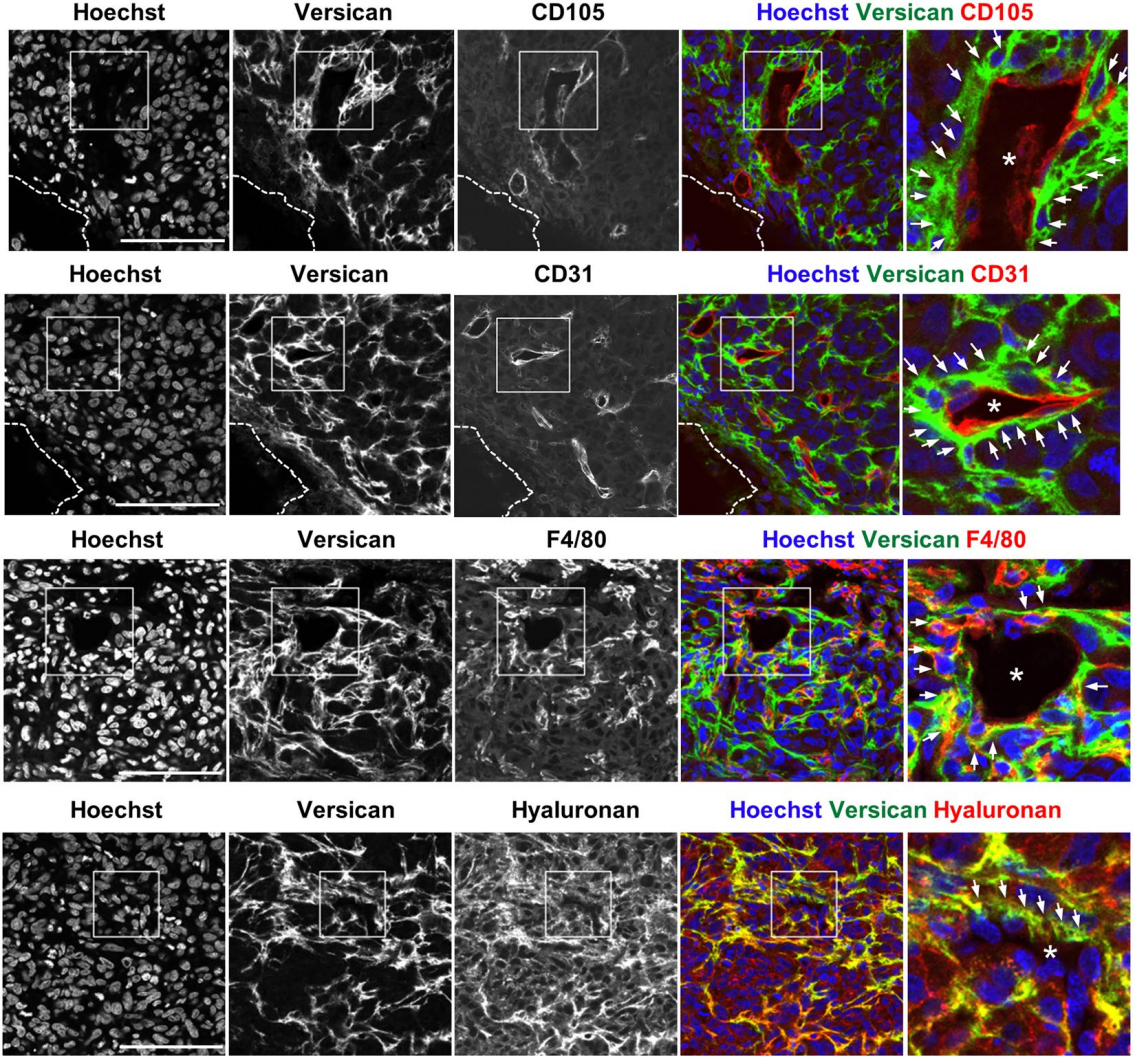
Localization of versican GAGβ in the vicinity of MDA-MB231 tumor vasculature. Immunofluorescence staining using anti-versican GAGβ, anti-CD31, anti-CD105, anti-F4/80 antibodies and biotinylated-hyaluronan binding protein (HABP) to compare the distribution of versican GAGβ (versican V0 and/or V1, green) with that of endothelial cells (CD105, CD31), macrophages (F4/80) or hyaluronan (each red) in the MDA-MB231 tumor. The arrows indicate overlapping localization of versican GAGβ with the vasculature, macrophages or hyaluronan, respectively. Nuclei were stained with Hoechst dye (blue). The white dashed lines indicate the edge of each tumor. Asterisks indicate the vessel-like structures. Scale bars = 100 μm.

We co-stained for versican GAGβ and two endothelial markers, CD105, and CD31. This demonstrated that versican primarily localized to the vicinity of the vasculature in the MDA-MB231 tumors. However, the endothelial cells themselves, which were stained with CD105, and CD31, did not co-stain for versican (Fig. 2A). Since recent studies have demonstrated that macrophages are essential for tumor angiogenesis^41,42^, we examined the localization of macrophages (F4/80^+^ cells) in relation to versican. Versican GAGβ staining showed co-localization with macrophages in the vicinity of the vasculature. Our results suggested that macrophages which infiltrated to the tumor tissues may produce the stromal-derived versican or may bind to versican-rich ECM (Fig. 2). Because versican aggregates with HA in various tissues, we combined biotinylated HA-binding protein (HABP) and anti-versican GAGβ staining which revealed their co-localization in the MDA-MB231 tumor stroma (Fig. 2). The specificity of the HA association was demonstrated by staining of sections with or without prior hyaluronidase treatment (Supplementary Fig. S5). A versican-HA complex is therefore a component of the tumor stroma. Furthermore, in association with macrophages, versican-HA complex was specifically localized to the vasculature which permeates solid tumors.

### Cleaved versican is observed in the tumor vasculature

**Figure 3:**
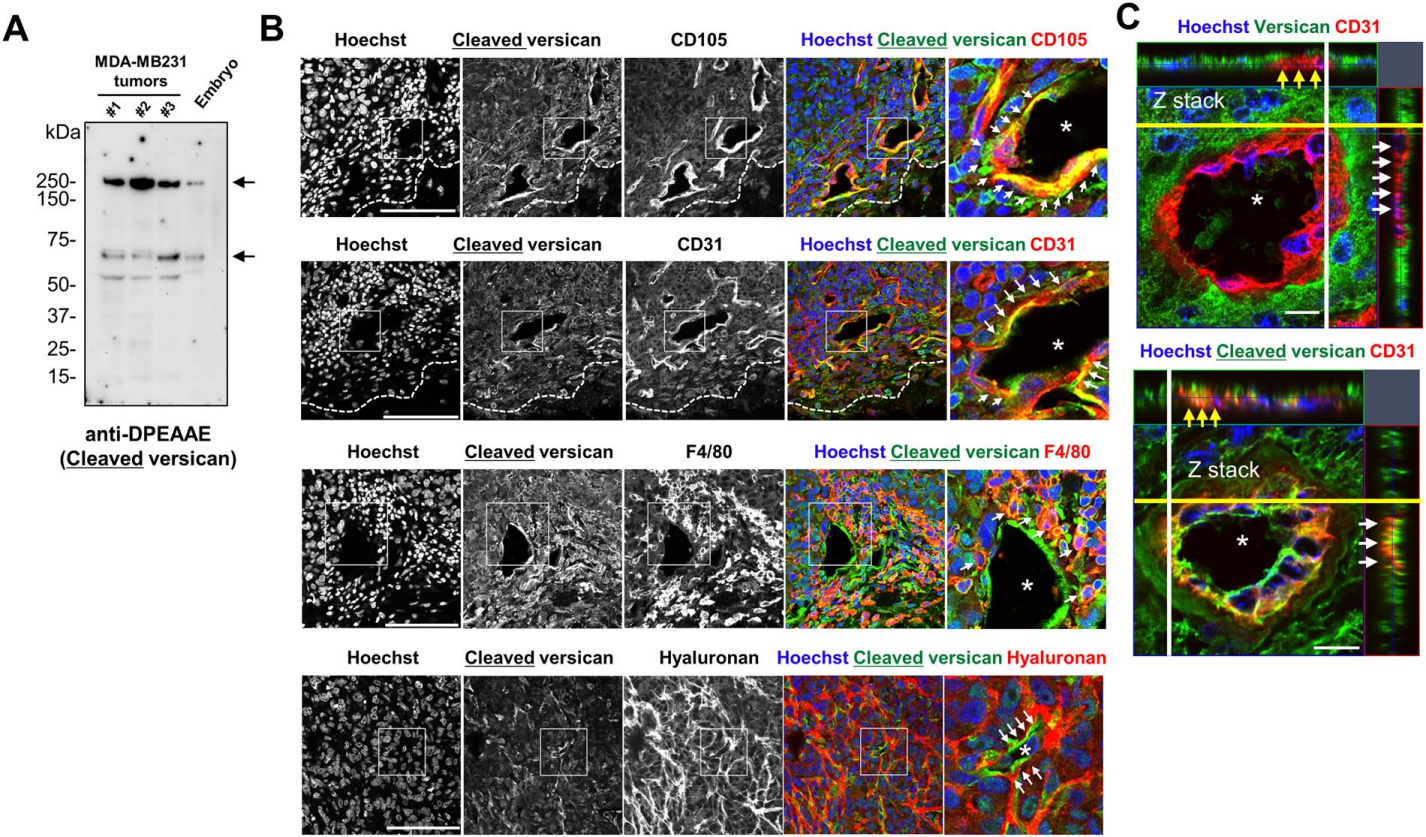
ADAMTS-cleaved versican in the MDA-MB231 tumor vasculature. (**A**) Western blot analysis with anti-DPEAAE antibody for ADAMTS-cleaved versican in the MDA-MB231 tumor extract. Protein extract from an E13.5 mouse embryo (Embryo) was used as the positive control. Arrows indicate the predicted size of cleaved versican (220 kDa presumably arising from V0, 70 kDa from V1). (**B**) Immunostaining using anti-DPEAAE, anti-CD31, anti-CD105 anti-F4/80 antibodies and biotinylated hyaluronan-binding protein (HABP) compared localization of cleaved versican with that of vasculature, macrophages or hyaluronan. Arrows indicate some overlap of cleaved versican in endothelium and macrophages and show dissociation of cleaved versican from hyaluronan distribution. (**C**) Z-stack confocal imaging comparing the versican GAGβ (intact versican) with cleaved versican in the vascular endothelium. Cleaved versican overlapped with endothelium stain while the intact versican did not (arrows). Asterisks indicate capillaries. Nuclei were stained with Hoechst dye (blue). Scale bars = 100 μm in **A**, **B**; 10 μm in **C**.

Cleavage of versican has been observed in vascular inflammation and pathological angiogenesis^31,43^. We analyzed ADAMTS-cleaved versican using anti-DPEAAE, which specifically detects the neoepitope resulting from proteolysis of versican at the Glu^441^-Ala^442^ site (V1 sequence enumeration)^44^. Western blotting detected both the expected 220 kDa and 70 kDa band in the MDA-MB231 tumors indicating cleavage of V0 and V1 respectively by ADAMTS proteases^45,46^. An additional 53 kDa anti-DPEAAE reactive band that presumably arises by cleavage within the G1 domain was observed. These findings indicated ADAMTS proteolytic activity in the tumor (Fig. 3A). Immunostaining with anti-DPEAAE, which stained E13.5 mouse embryo interdigital tissue as a positive control (Supplementary Fig. S3B) showed staining at the periphery of MDA-MB231 tumors as well as in vascular strands permeating the tumors (Fig. 3B). Overlapping staining with anti-GAGβ was thus seen, but in contrast to versican GAGβ, anti-DPEAAE signal was specifically localized to CD31 and CD105 positive cells within MDA-MB231 tumors (Fig. 3B,C).

**Figure 4:**
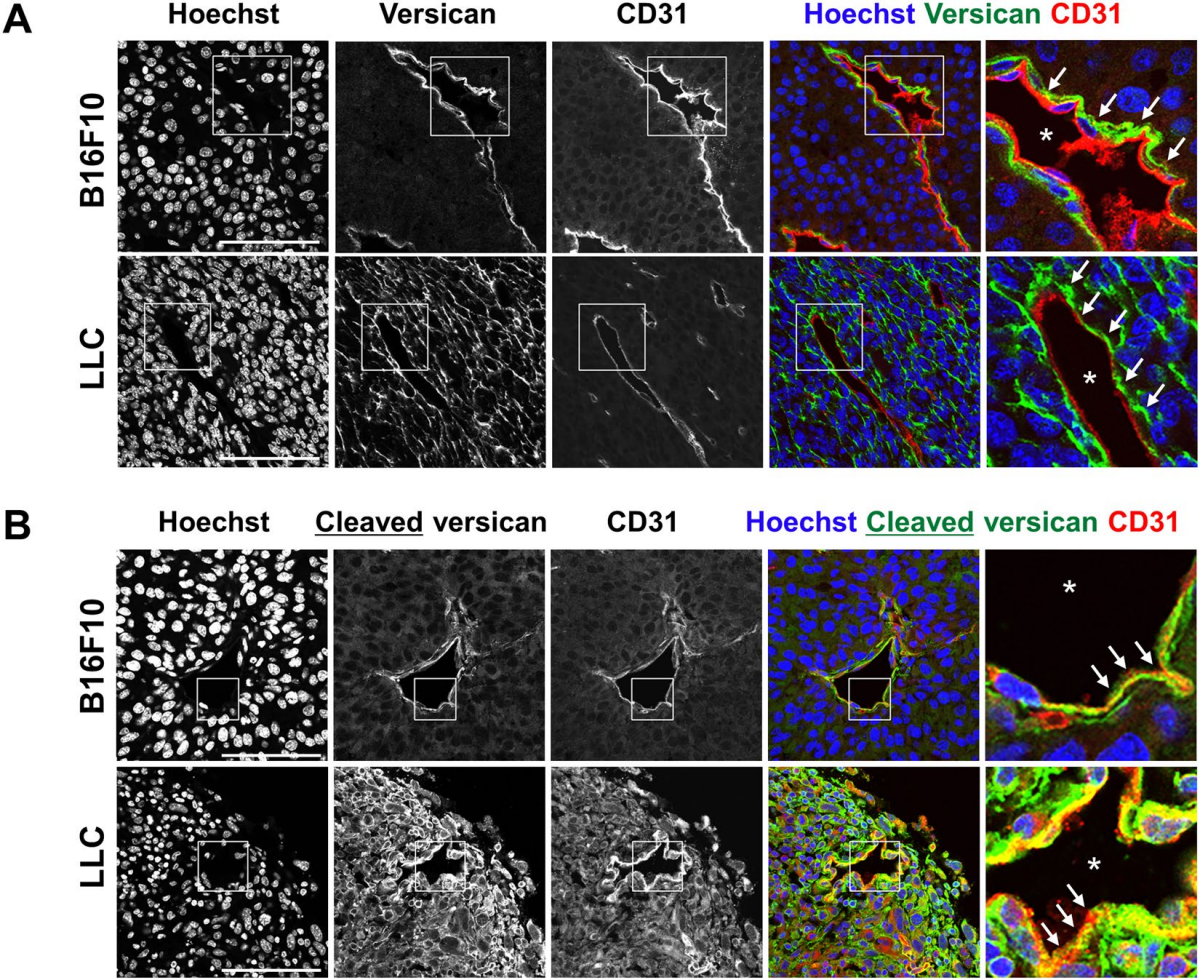
Versican and versikine localization in the B16F10 and LLC tumor vasculature. (**A**) Immunofluorescence staining using anti-versican GAGβ or anti-DPEAAE (green), anti-CD31 (red), or Hoechst nuclear dye (blue) as indicated to compare the distribution of versican GAGβ (versican V0 and/or V1) or versikine (anti-DPEAAE) with that of endothelial cells (CD31). Boxed areas in gray-scale images are shown at higher magnification and in color. The arrows indicate localization of versican GAGβ or versikine in the vasculature. The vascular lumen is indicated by the asterisk. Scale bars = 100 μm.

Furthermore, anti-DPEAAE signal in endothelial cells was distinct from HABP staining (Fig. 3B) although anti-DPEAAE showed distinct overlap with macrophages labeled by F4/80 (Fig. 3B) as we observed in GAGβ staining. Similar versican distribution, that is, positive signals closely associated with the vasculature, was observed in B16F10- and LLC-tumors and cleaved versican was similarly specifically associated with the endothelial cells (Fig. 4A,B). These results indicated that cleaved versican relocated to endothelial cells after cleavage of versican in the perivascular stroma.

### Genetic reduction of versican attenuates tumor angiogenesis and reduces tumor growth

**Figure 5:**
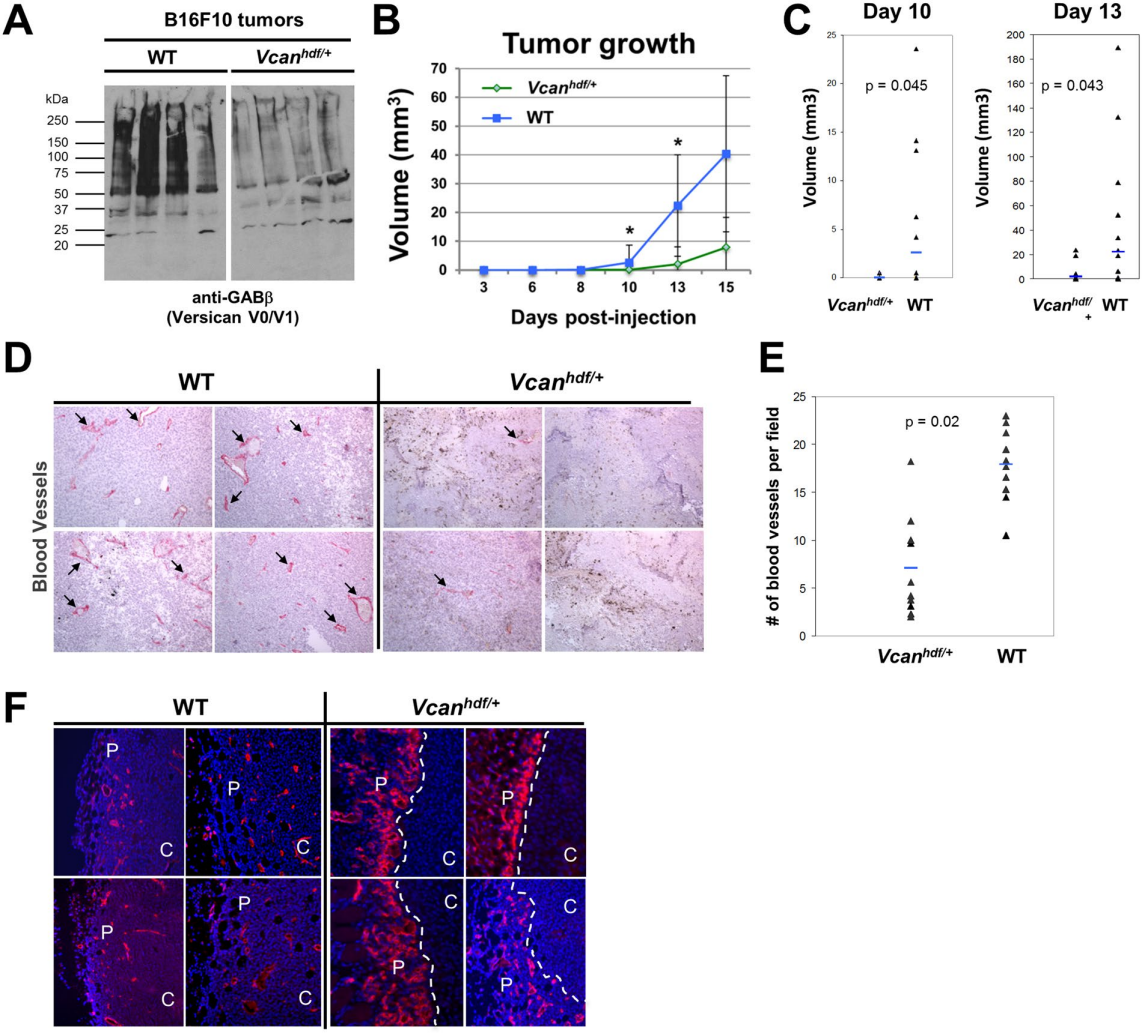
Impaired B16F10 tumor growth and angiogenesis in *Vcan* ^*hdf/*+^ mice. (**A**) Western blotting of tumors obtained from wild-type (WT) and *Vcan* ^*hdf/*+^ mice consistently showing reduced versican (using anti-GAGβ) in the latter. Originally, the Western was performed on a single membrane and the extra lane in the middle was excised. (**B**) Comparison of tumor growth rate in wild-type and *Vcan* ^*hdf/*+^ mice. Asterisks indicate the times after injection when tumor volume was statistically significant. n = 15 for each experiment. (**C**) Dot plots reporting tumor volumes in wild-type and *Vcan* ^*hdf/*+^ mice. (**D**) Immunostaining of the tumor interior with anti-endomucin antibody (red) to identify capillaries (arrows). Sections were counterstained with hematoxylin. (**E**) Vessel counts obtained from wild-type and *Vcan* ^*hdf/*+^ mice. n = 10 for each experiment. (**F**) Immunostaining of tumor vasculature using anti-endomucin (red). Nuclei were stained blue using Hoechst dye. White dashed lines *Vcan* ^*hdf/*+^ timors indicates an abrupt boundary restricting most vasculature to the periphery (P); C, center.

To determine the influence of host versican on tumor angiogenesis, we compared tumor growth and tumor angiogenesis in B16F10 tumors formed in *Vcan* ^*hdf/*+^ mice (haploinsufficient for versican), and wild-type littermates. B16F10 cells were chosen since our analysis indicated that these cells expressed little versican even when they formed tumors. Thus we could determine the host contribution in isolation. By immunoblotting, we first confirmed versican GAGβ reduction in *Vcan* ^*hdf/*+^ mouse tumor as compared with that of wild-type (Fig. 5A). We observed a significant reduction of tumor volume in the *Vcan* ^*hdf/*+^ mice at 10 days and 13 days post-injection compared to wild-type mice (Fig. 5B,C). In addition, B16F10 tumors in *Vcan* ^*hdf/*+^ mice had fewer capillaries than wild-type (Fig. 5D,E). Interestingly, we observed a high capillary density at the periphery of tumors in *Vcan* ^*hdf/*+^ mice, with few capillaries penetrating into the center, suggesting impaired vascular invasion into the tumor interior (Fig. 5F). Thus, these results suggest that host-derived versican, or cleaved versican facilitated tumor growth and angiogenesis.

## Discussion

**Figure 6:**
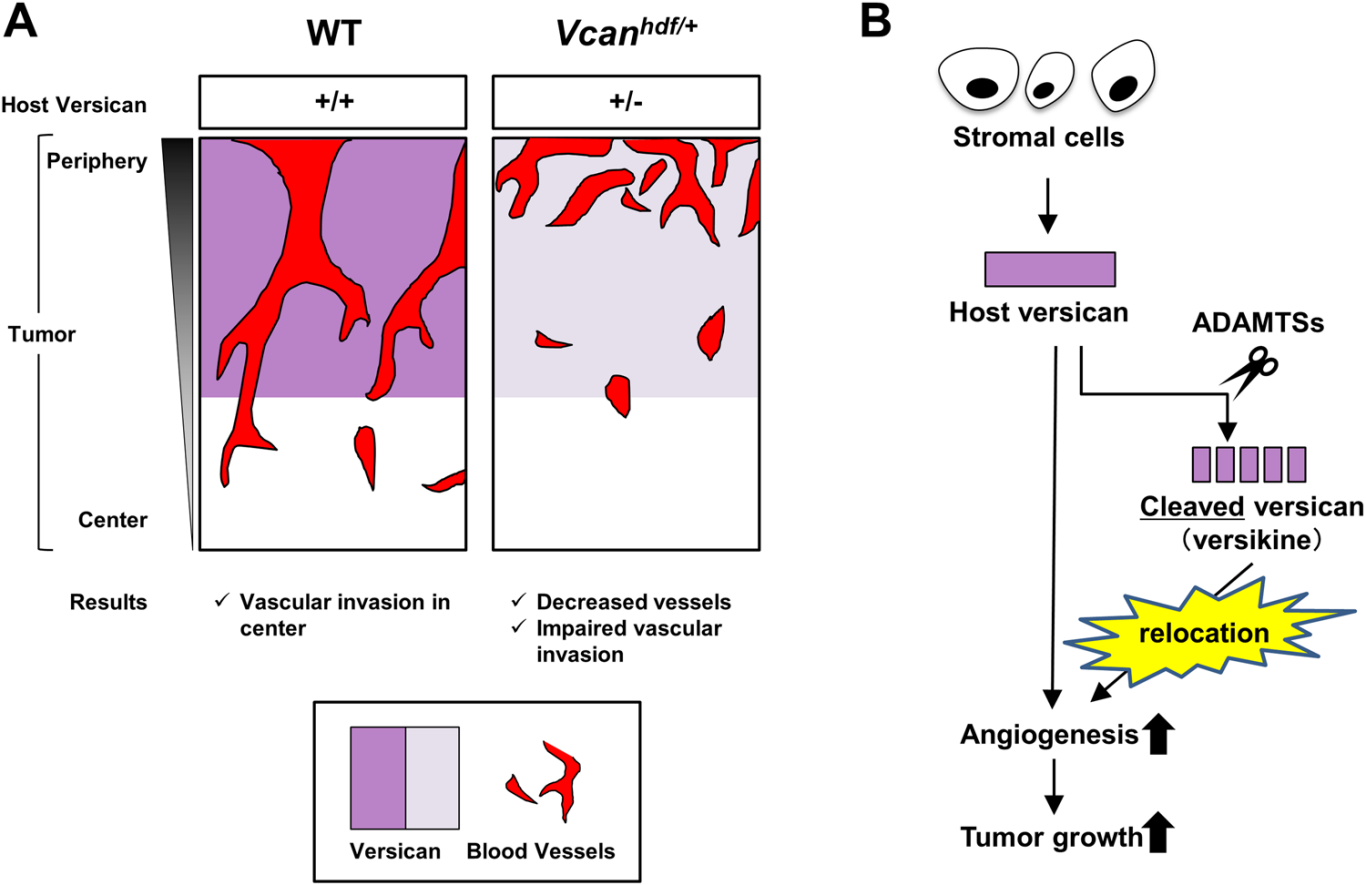
Proposed models for host-derived versican contribution for tumor development. (**A**) Cartoon showing impaired vascular invasion in tumors growing in *Vcan* ^*hdf/*+^ mice and accumulation of vasculature at the tumor periphery in *Vcan* ^*hdf/*+^ mice. (**B**) A schematic outlining the suggested role of versican in the tumor stroma and relocation of cleaved versican (versikine) to the endothelial cells.

These studies provide a number of new insights. We show that tumor cell lines, which have not been previously systematically studied in this manner, differ considerably in their versican expression levels. Previous studies have mostly focused on the versican expressed by tumor cells or not attempted to distinguish between stromal and cancer cell versican expression^47–49^. Our findings suggest that, in addition to the tumor-derived versican, the stroma-derived versican is extremely relevant in specific cancer models (Fig. 6). We demonstrated that in all the tumors investigated, versican of host (i.e., stromal origin) was distributed in the tumor periphery and associated with the tumor vasculature and its connective tissue that invades the tumor. In addition, the distribution of ADAMTS-protease cleaved versican showed a relocation of the N-terminal G1-domain-containing versican fragment, also called versikine, as compared with intact versican. A previous report showed that in limb bud development, cleaved versican had a different distribution from that of intact versican in association with apoptosis in interdigital tissue^34^. The present results were in line with the previous results, and suggests an independent role of versikine, a proteolytically generated versican fragment (Fig. 6). Finally, the studies show that tumorigenesis and angiogenesis were both impaired in *Vcan* haploinsufficient mice.

Another significance of this work relates to the frequent association of high tumor versican levels with higher cancer grade and adverse outcome^50^. Our studies suggest that where tumor-derived versican is present in addition to stromal versican there will be higher overall versican levels. Our studies also suggest that this is likely to contribute to accelerated vascular invasion and/or angiogenesis, which would enhance tumor growth. The present findings are not in agreement with a prior study in which tumorigenesis was undertaken using a versican-negative fibrosarcoma cell line and adenovirus Cre-induced stromal Vcan inactivation^51^. In contrast to the present analysis, the authors of that study concluded that stromal versican was inhibitory to angiogenesis and tumor growth. The contrasting findings could reflect different tumor and mouse models used in our respective approaches.

ADAMTS proteases have a major role in versican turnover during morphogenesis^9^. Our results suggest that ADAMTS proteases, such as ADAMTS-1, -4, -5 and -9 arising from endothelial cells and known to cleave versican^37^ may act in the perivascular region, and the cleaved versican may be relocated to endothelial cells through as yet unknown mechanisms. Overexpression, silencing or mutation of ADAMTS proteases has been reported in a variety of cancers^52^, among them the versican-degrading metalloproteases ADAMTS-1, -4, -5, -9 and -20^32,53–55^. These proteases may well have other roles in cancer through cleavage of additional substrates and thus the role of ADAMTS proteases in cancer progression needs to be further investigated. In addition, relocation of versikine to endothelial cells and reduced angiogenesis upon *Vcan* haploinsufficiency hints at a biological effect of versikine on angiogenesis requiring further investigation. It is interesting that many extracellular matrix protein fragments (termed matrikine or matricryptins) arising from proteolytic cleavage have been implicated in angiogenesis and other processes^56–58^.

Because several ADAMTS proteases can degrade versican, not only one particular ADAMTS, but ADAMTS proteases working in combination and with other protease classes may cooperate in complex patterns of versican fragmentation that need to be investigated. Indeed, each of the tumor Western blots showed extensive versican fragmentation. Because versican haploinsufficiency impaired proper tumor vessel invasion, it will be important to discriminate between the roles of intact and cleaved versican. Future studies using mice with cleavage-resistant versican or treatment with recombinant versikine could resolve these questions. Taken together, our findings suggest that versican is an intriguing target to consider in combating tumor growth and angiogenesis.

## Materials and Methods

### Mouse strains and cell lines

All animal experiments were performed in accordance with relevant guidelines and regulations and approved by institutional committees at Okayama University (#OKU-2016 500, #OKU-2017 174 and #OKU-2017 175) and the Cleveland Clinic (Protocol 2013-1137). C57BL/6 wild-type mice and BALB/c nude mice were purchased from Charles River Japan, Yokohama, Japan. *Vcan* ^*hdf/*+^ mice^59^ were obtained under a material transfer agreement with Roche Pharmaceuticals and were maintained by outcrossing to C57BL/6 mice (Jackson Laboratories, Bar Harbor, ME, USA). MDA-MB231 human breast tumor cell line and the B16F10 melanoma cell line were obtained from American Type Culture Collection (ATCC, Rockville, MD, USA) and mouse LLC was purchased from European Collection of Authenticated Cell Cultures (ECACC, Public Health England, UK)^60^. Normal human skin fibroblasts (HSF) were purchased from Lonza Japan (Tokyo, Japan) and cultured with fibroblast basal growth medium-2 BulletKit and cells prior to passage 10 were used for analysis as previously described^61^. MDA-MB231, B16F10 and LLC cells were cultured in Dulbecco’s modified Eagle’s medium (DMEM) with 10% fetal bovine serum (FBS), 100 U/mL penicillin and 100 μg/mL streptomycin. Cells were cultured at 37 °C under 20% O_2_ and 5% CO_2_ condition in a humidified incubator.

### RNA extraction, RT-PCR and qPCR

To determine gene expression, 2 × 10^5^ MDA-MB231, B16F10, LLC cells and HSF were seeded in 12 well plates and cultured for 24–48 hours. Total RNA was extracted using TRIzol (Thermo Fisher Scientific, Waltham, MA, USA), as previously described^62,63^. Briefly, B16F10 and LLC tumors, mouse aorta and brain were homogenized with TRIzol for total RNA extraction according to the manufacturer’s protocol. The RNA was kept at -80 °C until used for the experiments. The RNA concentration was measured using NanoDrop (Thermo Fisher Scientific) and 2 μg/20 μL of RNA was used for reverse transcription (ReverTra Ace, Thermo Fisher Scientific) at 30 °C for 10 minutes, 42 °C for 60 minutes and at 85 °C for 10 minutes as previously described^64,65^. Human brain cDNA was obtained by reverse transcription of human brain total RNA (Thermo Fisher Scientific). The cDNA was diluted five-fold prior to PCR. RT-PCR was performed using rTaq DNA polymerase (Takara Bio Inc., Kusatsu, Japan) with specific primers^66^. Primers used for RT-PCR were shown in Supplementary Table S1. Amplified products were electrophoresed on 2% agarose gels. For qPCR, 1 μL cDNA was mixed with TaqMan Fast Advanced Master Mix and specific primer pairs for *VCAN* V0 and V1 isoforms and *Vcan* V0 and V1 mRNAs (Thermo Fisher Scientific) according to the manufacturer’s protocol. Gene expression was normalized using *RPLP2* (for human) and *Gapdh* (for mouse) primers respectively. TaqMan primers used for qPCR were; *VCAN* V0, Hs01007944_m1; *VCAN* V1, Hs01007937_m1; *RPLP2*, Hs01115128_gH; *Vcan* V0/V1, Mm00490174_m1; mouse *Gapdh*, Mm99999915_g1. Gene expression was analyzed by ∆∆Ct method as previously described^67–69^. All experiments were repeated at least three times independently.

### Tumor-bearing mouse models

1.0 × 10^6^ MDA-MB231 cells suspended in 100 μL of DMEM were injected subcutaneously in BALB/c nude mice (7–10 week old female) as previously described^70,71^. Similarly, B16F10 and LLC cells were implanted in the skin of C57BL/6 mice (including *Vcan* ^*hdf/*+^ and wild-type littermates; 7–10 week old female). Tumor growth was measured with a Vernier caliper every two or three days. Tumor volume was determined by the formula (volume = length × width^2^ × 0.52). Mice were euthanized when or before the tumor size reached 500–1000 mm^3^ and the tumor tissues were dissected for following experiments.

### Immunohistochemistry

Tumors and E13.5 limb buds were fixed with 4% paraformaldehyde (PFA) for 24–48 hours at 4 °C and used for frozen sections or were paraffin-embedded. For frozen sections, tumors were treated with 30% sucrose and immersed in OCT compound for freezing in liquid nitrogen prior to sectioning with a cryostat. Samples were treated with 0.05 U/mL chondroitinase ABC (Sigma-Aldrich, St. Louis, MO, USA) with 0.03 M NaOAc buffer and 0.1 M Tris HCl pH 8.0 buffer for 1 hour at 37 °C prior to antibody reaction. For inhibition of non-specific binding, tissues were incubated with 5% skim milk diluted with PBS for 1 hour at room temperature and then treated with primary antibody overnight at 4 °C. Primary antibodies used for immunohistochemistry were anti-rabbit versican GAGβ (ab1033, Millipore, Billerica, MA, USA, 1:100); anti-DPEAAE (ab19345, Abcam, Cambridge, UK, 1:100); anti-CD31 (557355, BD Biosciences, San Jose, CA, USA, 1:200); anti-CD105 (550546, BD Biosciences, 1:200); and anti-F4/80 (MCA497GA, BioRad, Hercules, CA, USA, 1:100). Rat anti-endomucin monoclonal antibody (ab106100, Abcam) was used to identify capillaries. Secondary antibodies used for immunohistochemistry were anti-rabbit IgG Alexa 647 (A21443, Thermo Fisher Scientific, 1:500); anti-rat IgG Alexa 488 (A11006, Thermo Fisher Scientific, 1:500); and anti-Streptavidin conjugated Alexa 488 (S11223, Thermo Fisher Scientific, 1:500). Nuclei were stained with Hoechst dye (Sigma-Aldrich) before coverslip mounting for immunofluorescence and confocal laser scanning microscopy (model LSM780, Zeiss, Oberkochen, Germany) at the Central Research Laboratory, Okayama University Medical School. To detect HA, tissues were treated with HABP (2 μg/mL concentrations, Hokudo, Sapporo, Japan) overnight at 4 °C then reacted with Alexa Fluor 488 streptavidin conjugate. For the HA digestion, tissues were pretreated with 0.1 M sodium acetate pH 6.0 buffer for 15 minutes at 37 °C and then treated with 10 Turbidity Reducing Unit (TRU) /mL of *Streptomyces hyalurolyticus* hyaluronidase (Amano Enzyme, Nagoya, Japan) with the buffer for 2 hours at 60 °C prior to the HABP reaction. All experiments were repeated at least three times, independently.

### RNA *in situ* hybridization

For *in situ* hybridization, we used the RNAScope method (Advanced Cell Diagnostics, Newark, CA, USA) with specific probes targeting *VCAN* exon 7 (452231 Red) or exon 8 (452241 Red) (encoding the GAGα and GAGβ domains respectively) or *Vcan* exon 7 (428311 Red) or exon 8 (428321 Red). As a control for versican RNA expression, human umbilical cord was obtained under approval from the Cleveland Clinic Institutional Review Board with an exemption (exemption 4) for the use of discarded human tissue without patient identifiers. 7 µm sections were cut immediately prior to *in situ* hybridization, de-paraffinized and hybridized to the probes using the RNAScope 2.0 HD Red detection kit and HybEZ^TM^ oven (Advanced Cell Diagnostics) according to the manufacturer’s instructions. Sections were stained with hematoxylin and then treated with ammonium hydroxide to acquire blue counter staining as previously described^72^.

### Western blotting

5 × 10^5^ MDA-MB231-, B16F10- and LLC-cells were independently seeded on 6-well plates and cultured for 24 hours. Culture medium was replaced with FBS-free medium and then cultured for a further 24 hours. Conditioned media were filtered with 0.22 μm pore filter and centrifuged and supernatant was stored at −80 °C. Cells were lysed with CelLytic M Mammalian Cell Lysis/Extraction Reagent (Sigma-Aldrich) and centrifuged. The MDA-MB231 xenograft tumors were homogenized with lysis buffer (T-PER Tissue Protein Extraction Reagent, Thermo Fisher Scientific) and centrifuged as previously described. Proteinase inhibitor (Roche, Basel, Switzerland) was added to the lysis buffer to protect protein degradation. Protein concentration was measured using Pierce BCA Protein Assay kit (Thermo Fisher Scientific), according to the manufacturer’s instruction. For the detection of versican GAGβ, chondroitinase digestion was performed as described above. Protein extracted from MDA-MB231 tumor (30 μg), E13.5 mouse embryo (15 μg) and cell lysates (15 μg) was used for Western blot analysis, as described previously^73,74^. The primary antibodies were anti-versican GAGβ (as described above); anti-neoepitope DPEAAE (as described above); anti-β-actin (A5441, Sigma-Aldrich, 1:5000); and secondary antibodies for anti-rabbit IgG HRP conjugated (55676, MP Biomedicals, Santa Ana, CA, USA, 1:2000); and anti-mouse IgG HRP conjugated (sc-2005, Santa Cruz, Dallas, Texas, USA, 1:2000) were used and developed by using Amersham ECL Prime (GE Healthcare, Buckinghamshire, England, UK). All experiments were repeated at least three times, independently.

### Statistical analysis

All values are given as the mean ± S.D. Between-group variations were assessed using the two-tailed unpaired t-test. For multiple comparisons, analysis of variance was performed, with post-hoc analysis with the Bonferroni test. P values < 0.05 were considered significant.

## Electronic supplementary material

Supplemental data
