# Supplementary material for: Stromal Versican Regulates Tumor Growth by Promoting Angiogenesis
Source: Sci Rep. 2017 Dec 8;7:17225. doi: 10.1038/s41598-017-17613-6 (PMC5722896; doi:10.1038/s41598-017-17613-6)
Supplement: Supplementary file 1 — Supplemental data [file 41598_2017_17613_MOESM1_ESM.pdf]

STROMAL VERSICAN REGULATES TUMOR GROWTH BY PROMOTING ANGIOGENESIS

Keiichi Asano[1,5], Courtney M. Nelson[2], Sumeda Nandadasa[2], Noriko Aramaki-Hattori[2], Daniel J. Lindner[3], Tyler Alban[2], Junko Inagaki[4], Takashi Ohtsuki[5], Toshitaka Oohashi[1], Suneel S. Apte[2], Satoshi Hirohata[5]*

Supplementary Figures and Table

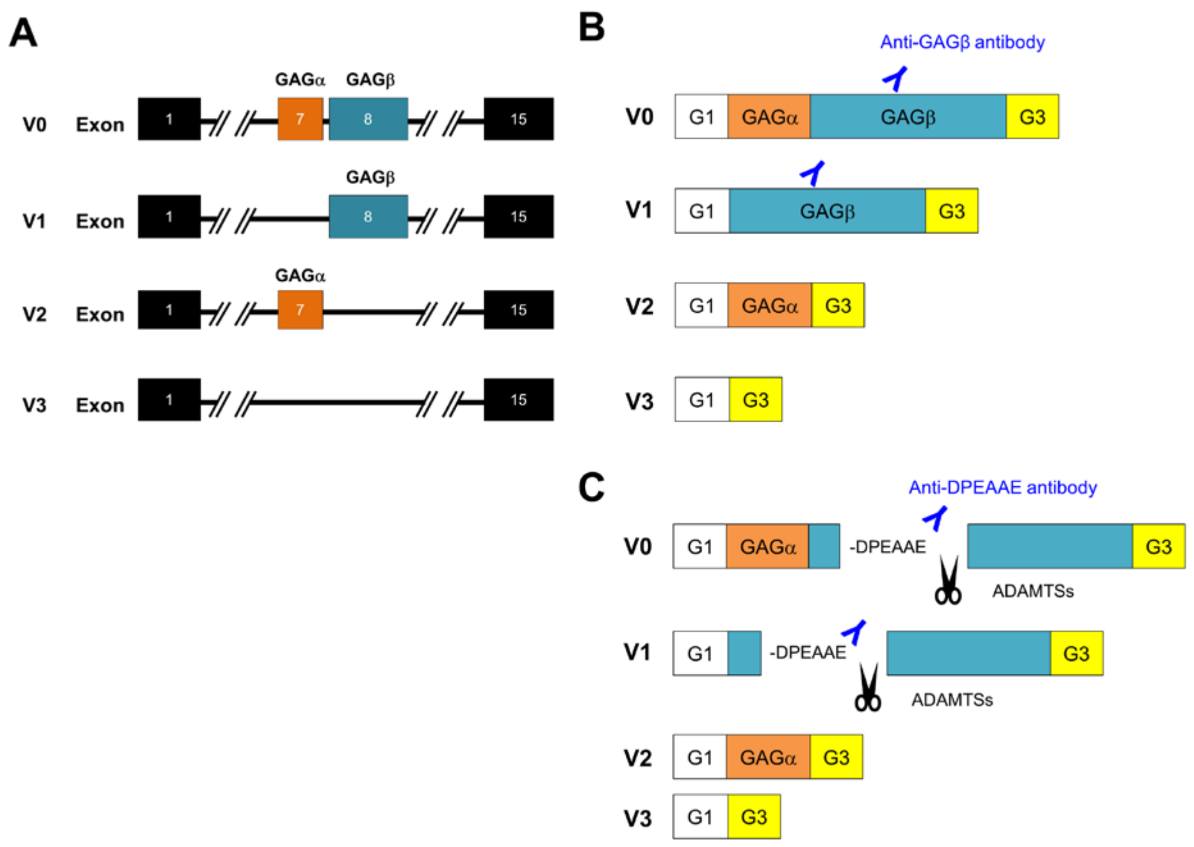

Supplementary Figure S1. Schematic of versican splicing versican antibody epitopes

(A) The versican gene contains 15 coding exons. Exon 7 and exon 8 encodes GAGα and GAGβ domains respectively. The splicing of these exons to generate the various isoforms is shown **(B)** Domain structure of each versican isoform. Anti-versican GAGβ antibody recognizes both versican V0 and V1 isoforms but not V2 and V3. **(C)** Versican-cleavage by ADAMTS proteases and its recognition by anti-DPEAAE neoepitope antibody. Versican V0 and V1 isoforms are cleaved by ADAMTS protease at a specific site in the GAGβ domain, Anti-DPEAAE antibody specifically recognizes a neoepitope revealed by ADAMTS protease activity. .

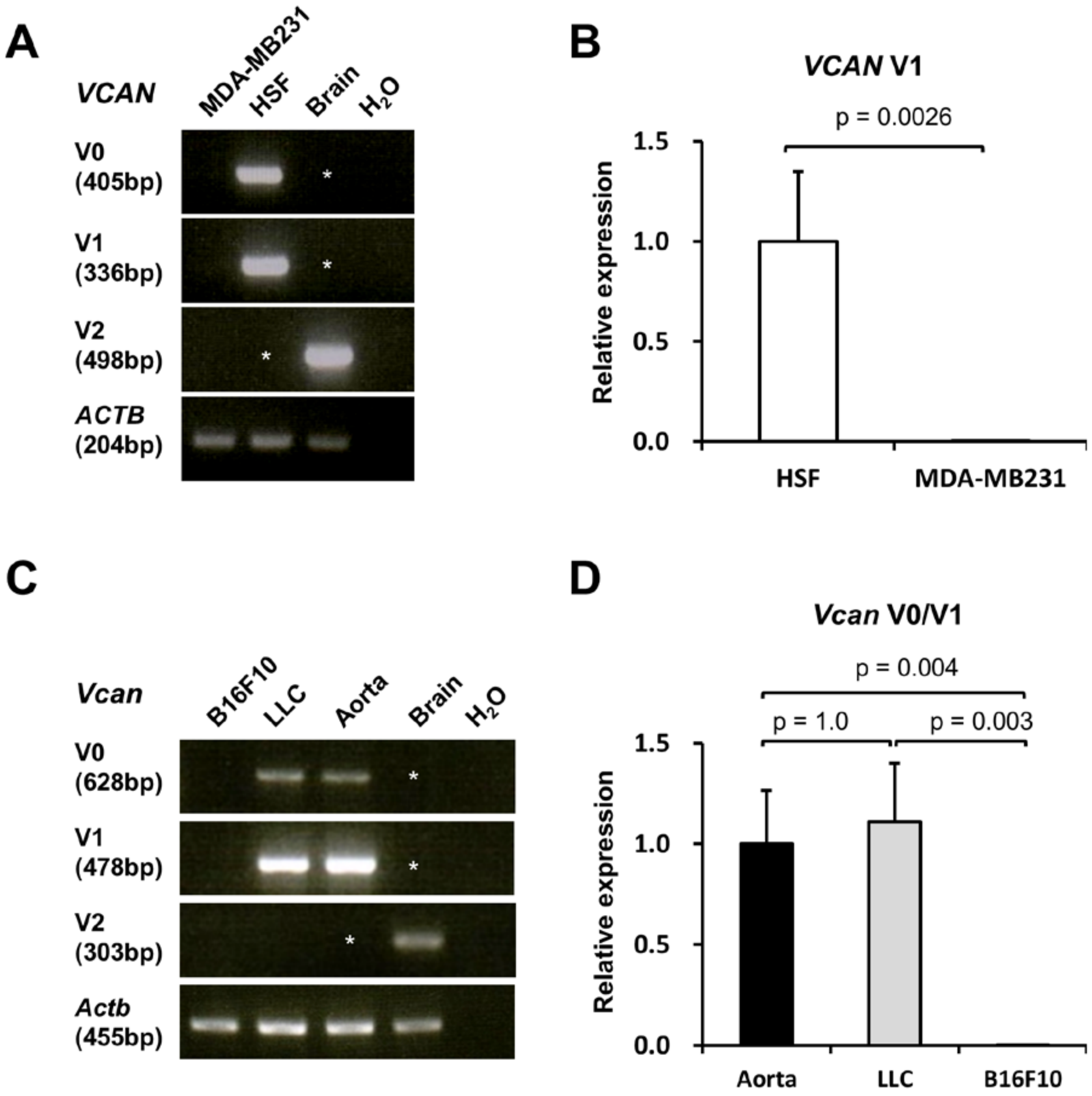

Supplementary Figure S2: Variable expression of versican isoforms in human- and mouse-tumor cell lines. Expression of versican isoforms in MDA-MB231 human breast tumor cell line and in B16F10- and Lewis lung carcinoma (LLC)-mouse tumor cell lines was determined by reverse transcription PCR (RT-PCR) and quantitative RT-PCR (qRT-PCR). 2× 10^5 MDA-MB231, B16F10, LLC cells and human skin Fibroblasts (HSF) were seeded in 12 well plates and cultured for 24-48 hours until these cells were confluent. Total RNA was extracted by using TRIzol and 2 μg/20 μL of RNA was reverse transcribed. (**A, C**) RT-PCR

using isoform-specific *VCAN* and *Vcan* primer listed in Supplemental Table 1. The asterisk indicates no amplification. The expression of versican was compared with positive controls; human skin fibroblasts (HSF) for *VCAN* V0 and V1; human brain for *VCAN* V2; mouse aorta for *Vcan* V0 and V1; mouse brain for *Vcan* V2. (**B, D**) qPCR compared the expression of *VCAN* V1 in MDA-MB231 cells with that of HSF; *Vcan* V0 and/or V1 (*Vcan* V0/V1) in B16F10- and LLC-cells with that of aorta. The expressions were normalized with those of *RPLP2* for human; *Gapdh* for mouse, respectively. For statistical significance, *=$p < 0.05$. n = 4 for *VCAN* qPCR; n = 3 for *Vcan* qPCR.

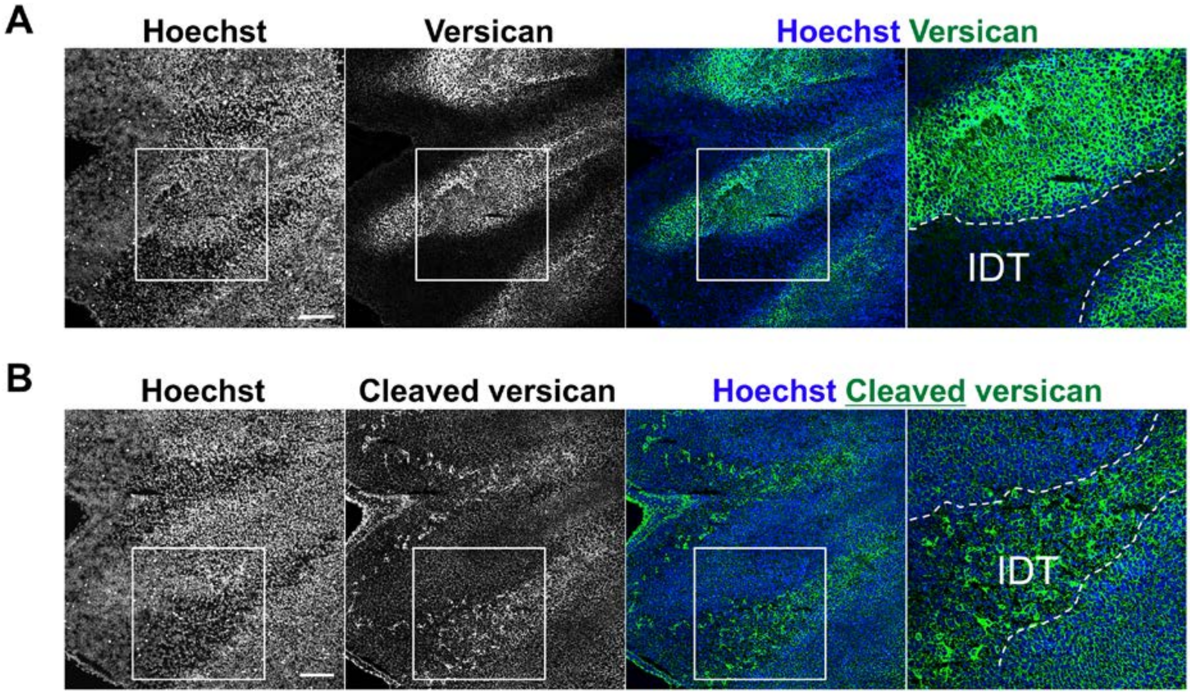

Supplementary Figure S3. Validation of specific staining for versican GAGβ and cleaved versican. Immunostaining using anti-versican GAGβ (green) (**A**) and anti-DPEAAE (neoepitope antibody) (green) (**B**) in E13.5 mouse forelimbs. Dashed lines indicate the border between digit cartilage and interdigital tissue (IDT). Nuclei were counterstained with Hoechst dye (blue). Scale bars = 100 μm.

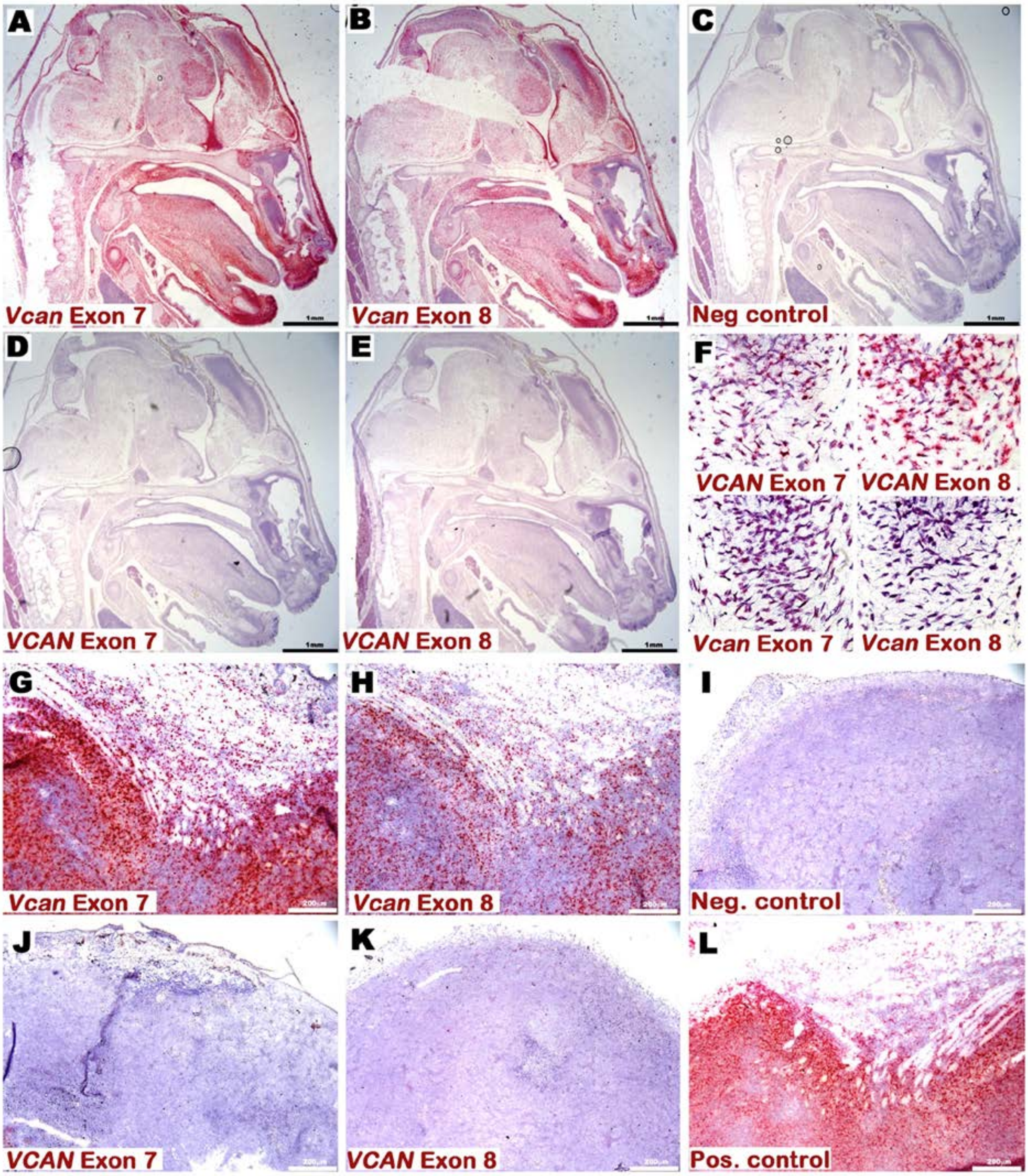

Supplementary Figure S4. Species-specificity of versican *in situ* probes. Cross reactivity of mouse-specific (*Vcan*) and human-specific (*VCAN*) *in situ* probes were determined using E16.5 wild type mouse embryo sections (**A-E**), human umbilical cord sections (**F**), and LLC tumors (**G-L**). Versican *in situ* probes generated against mouse exon 7 (GAGα) (**A**) and exon 8 (GAGβ) (**B**) showed robust *Vcan* expression (red) in mouse

embryonic tissues, while neither the negative control probe (**C**) nor the human exon 7- and exon 8-specific probes showed positive signal (**D-E**). The human exon 7 and exon 8 probes showed robust hybridization in smooth muscle cells of the human umbilical cord artery while the mouse specific probes did not show any hybridization (**F**). *In situ* hybridization of mouse *Vcan* probes in LLCs showed robust versican expression in tumor cells and stromal cells surrounding the tumors (**G-I**), which are not recognized by the human specific probes (**J-K**). Scale bars = 1mm in A-E, 200μm in G-L.

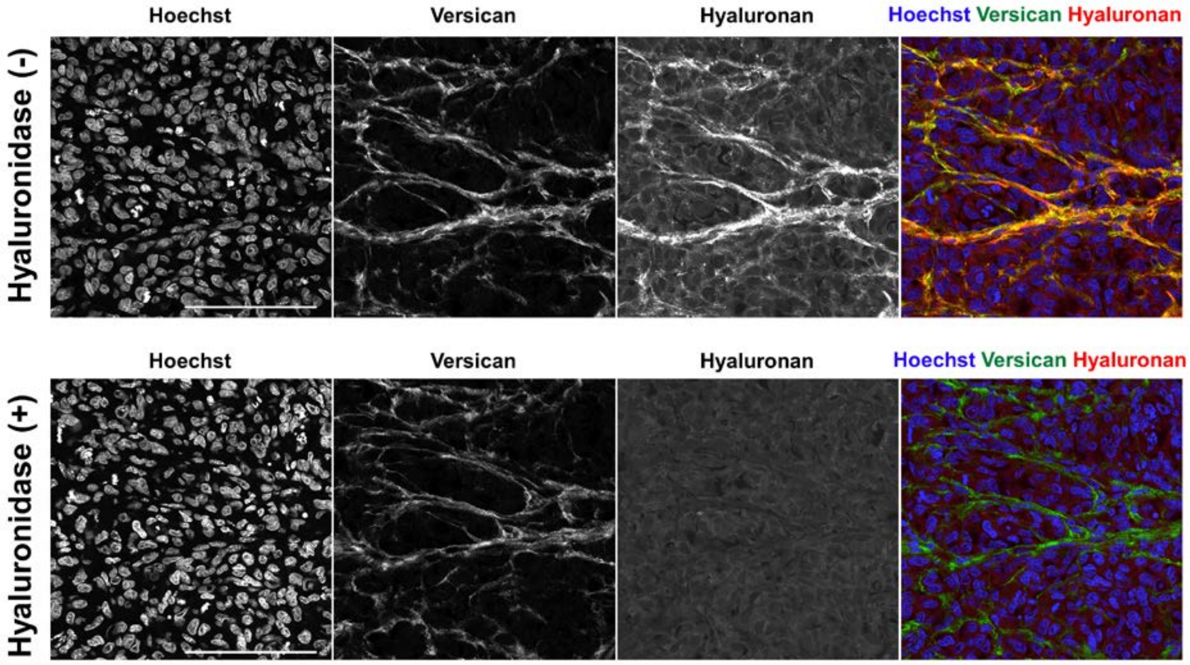

Supplementary Figure S5. Versican is co-localized with hyaluronan in MDA-MB231 tumors. Combined staining using anti-versican GAGβ antibody (green) and biotinylated-hyaluronan binding protein (HABP, red) determined the co-distribution of versican GAGβ and hyaluronan (upper panels). As a negative control, tissue was treated with hyaluronidase (lower panels), which abolished HABP binding. Nuclei were counter-stained with Hoechst dye (blue). Arrows indicate positive signals for each staining. Scale bars = 100 μm.

Supplementary Table S1: Primers used for RT-PCR

Target gene	Species	Forward	Reverse
Vcan V0	Mouse	5'-TTCACAGAACGCCACCCTTGAGTCC-3'	5'-CTAGCTTCTGCAGCTTCCGGGTCC-3'
Vcan V1	Mouse	5'-GCAGCTTGGAGAAATGGCTTTGACC-3'	5'-CTAGCTTCTGCAGCTTCCGGGTCC-3'
Vcan V2	Mouse	5'-TCCTGGAGAATCTGTAACACAGCACC-3'	5'-CTCGGTAGGATAACAGGTGCCTCCG-3'
Actb	Mouse	5'-TTCTACAATGAGCTGCGTGTGGC-3'	5'-CTCATAGCTCTTCTCCAGGGAGGA-3'
VCAN V0	Human	5'-TCAACATCTCATGTTCCTCCC-3'	5'-TTCTTCACTGTGGGTATAGGTCTA-3'
VCAN V1	Human	5'-GGCTTTGACCAGTGCGATTAC-3'	5'-TTCTTCACTGTGGGTATAGGTCTA-3'
VCAN V2	Human	5'-TCAACATCTCATGTTCCTCCC-3'	5'-CCAGCCATAGTCACATGTCTC-3'
ACTB	Human	5'-TTCCTGGGCATGGAGTCCT-3'	5'-AGGAGGAGCAATGATCTTGATC-3'
Vcan 3' UTR	Mouse	5'-GTGCTTCACTCATCATTTCAGC-3'	5'-AAAAGCCGTCCTCAAACTTGC-3'
VCAN 3' UTR	Human	5'-CAAAGTCCTAACTTCCTGTGC-3'	5'-CATTAAAGACCTCTAAGGCTCC-3'
